# Glucagon-Like Peptide-1 Receptor Agonists in Plastic Surgery: Perioperative Considerations and Safety Protocols

**DOI:** 10.7759/cureus.99865

**Published:** 2025-12-22

**Authors:** Rodrigo Davila Diaz, Eugenia Campos Barrera, Luis Alfonso Diaz Fosado, Arturo Reyes Esparza, Omar A Perez Benitez

**Affiliations:** 1 Plastic, Aesthetic, and Reconstructive Surgery, Hospital Angeles Pedregal, Mexico City, MEX; 2 Endocrinology, Diabetes, and Metabolism, Hospital Angeles Pedregal, Mexico City, MEX; 3 Anesthesiology, Hospital Angeles Pedregal, Mexico City, MEX; 4 Plastic, Aesthetic, and Reconstructive Surgery, Hospital Central Sur de Alta Especialidad de Petróleos Mexicanos, Mexico City, MEX

**Keywords:** elective plastic surgery, glp-1 ra, glucagon-like peptid-1 receptor agonists, perioperative considerations, safety in plastic surgery, safety protocols

## Abstract

Glucagon-like peptide-1 receptor agonists (GLP-1 RAs) are increasingly prescribed among plastic surgery patients for obesity and overweight. Agents such as dulaglutide, liraglutide, semaglutide, and tirzepatide differ in pharmacology and dosing, directly influencing perioperative safety, particularly due to their effects on gastric motility and the high frequency of gastrointestinal (GI) adverse events that raise concerns about aspiration and postoperative morbidity in urgent or elective procedures.

A narrative review was conducted using PubMed, Embase, and the Cochrane Library (2010-2025). Randomized controlled trials, meta-analyses, prospective studies, case reports, and perioperative guidelines were analyzed.

We reviewed each agent along with the administration route, half-life, average weight loss, and recommended discontinuation prior to surgery: dulaglutide: weekly injection, half-life of approximately five days, weight loss of 3%-5%, and discontinuation at >1 week before surgery; liraglutide: daily injection, half-life of approximately 13 hours, weight loss of 5%-7%, gastrointestinal (GI) adverse events up to 40%, and discontinuation at 24-48 hours before surgery; semaglutide (weekly): Efficacy and Safety of Once-Weekly Semaglutide Versus Exenatide Extended Release (ER) in Subjects With Type 2 Diabetes (SUSTAIN-3) showing superior glycated hemoglobin (HbA1c) reduction and weight loss compared with exenatide, GI adverse events including nausea (22%), diarrhea (11%), and vomiting (7%), and discontinuation at >1 week preoperatively; semaglutide (oral): Peptide Innovation for Early Diabetes Treatment (PIONEER) trials showing 4.4 kg weight loss at 26 weeks, GI adverse events of 15%-20%, and discontinuation at 24-48 hours prior; and tirzepatide: weekly dual glucose-dependent insulinotropic polypeptide/glucagon-like peptide-1 (GIP/GLP-1) agonist, weight loss of up to 20% in SURMOUNT-1, half-life of approximately five days, and discontinuation at >1 week before surgery.

Prospective ultrasound studies show a higher prevalence of residual gastric content in GLP-1 RA users, particularly during dose escalation or when GI symptoms are present. Case reports document perioperative aspiration in semaglutide users, including episodes of hypoxemia and reintubation despite guideline-concordant fasting.

Perioperative risk is now supported by prospective evidence and case reports. Weekly formulations show the strongest association with delayed gastric emptying and aspiration. Pharmacovigilance data and FDA/European Medicines Agency (EMA) alerts also describe rare cases of suicidal ideation and self-harm behaviors, especially in weight-loss therapy.

GLP-1 RAs are commonly used among plastic surgery patients, and their use has grown exponentially over the years. There are concerns about delayed gastric emptying and gastrointestinal intolerance that pose a perioperative challenge that demands proactive perioperative management by the plastic surgery team.

Plastic surgeons must lead the development of tailored, evidence-based, risk-stratified protocols that safeguard patients while preserving the benefits of these therapies. We share the protocol that we believe can help the plastic surgery team standardize safety strategies to be considered prior to elective surgery in patients who are treated with GLP-1 RA. More prospective studies are needed to validate and concentrate all guidelines and refine best practices in the era of metabolic pharmacology.

## Introduction and background

Glucagon-like peptide-1 receptor agonists (GLP-1 RAs) have revolutionized management in obesity and diabetes, showing better metabolic outcomes, and their use is increasingly common among plastic surgery patients [1-4]. Their mechanisms, such as delayed gastric emptying, appetite suppression, the enhanced feeling of satiety, and improved insulin regulation, explain both their therapeutic benefits and the perioperative risks they may pose [1,2,5-7]. Currently, there are no international consensus or guidelines in plastic surgery about patients who are treated with GLP-1 RA and are subjected to an elective or urgent plastic surgery.

The main agents currently in clinical use include the following: liraglutide: applied daily in a subcutaneous (SC) injection and a first-generation GLP-1 RA, which was approved for weight management in adults by the FDA in 2014; semaglutide: available as a weekly SC injection and a daily oral formulation; dulaglutide: available as a weekly subcutaneous injection; and tirzepatide: a dual glucose-dependent insulinotropic polypeptide/glucagon-like peptide-1 (GIP/GLP-1) receptor agonist administered as a weekly subcutaneous injection and a second-line type 2 diabetes medication with off-label use for obesity treatment.

GLP-1 binds to receptors on vagal afferent neurons and within the nucleus tractus solitarius, altering parasympathetic signaling that governs gastric tone and motility. This neural modulation decreases the activity of interstitial cells of Cajal and reduces acetylcholine release in the myenteric plexus, thereby slowing gastric transit [1-5,8-14].

Understanding the mechanism of action and pharmacokinetics, as well as the perioperative implications of these agents, is essential for plastic surgeons to design safe surgical protocols.

## Review

Pharmacology and posology

The pharmacokinetics of GLP-1 RA dictate perioperative management. Long-acting agents (semaglutide injectable, dulaglutide, and tirzepatide) require one to two weeks of discontinuation, while short-acting agents (oral semaglutide and liraglutide) are usually held for 24-48 hours, as seen in Table 1 [2,7-13,15,16].

**Table 1:** Pharmacological profile and perioperative discontinuation SC: subcutaneous

Drug	Half-life	Dosing	Formulation	Recommended discontinuation
Semaglutide injectable (Ozempic and Wegovy)	7 days	Weekly SC	High systemic and sustained	>1 week
Semaglutide oral (Rybelsus)	7 days (low bioavailability)	Daily oral	Lower and fluctuating	-
Liraglutide (Victoza and Saxenda)	13 hours	Daily SC	Moderate	-
Dulaglutide (Trulicity)	5 days	Weekly SC	High	>1 week
Tirzepatide (Mounjaro and Zepbound)	5 days	Weekly SC	Very high efficacy	>1 week

Methods

A narrative review was performed using PubMed, Embase, and the Cochrane Library (2010-2025). Search terms included “GLP-1 receptor agonists”, “semaglutide”, “liraglutide”, “dulaglutide”, “tirzepatide”, “perioperative management”, and “plastic surgery”. Randomized trials, phase II studies, meta-analyses, and anesthesia consensus guidelines were included. Evidence levels were graded according to the Oxford Centre for Evidence-Based Medicine.

Results

As part of our review, we found evidence from randomized trials, as well as plastic surgery-focused safety evidence, updated risk-stratified protocols, and comparisons of international guidelines, all of which develop and compare secondary effects of GLP-1 RA, perioperative anesthetic risk, and nutritional and psychiatric effects.

Evidence From Randomized Trials

The Efficacy and Safety of Once-Weekly Semaglutide Versus Exenatide Extended Release (ER) in Subjects With Type 2 Diabetes (SUSTAIN-3) trial by Ahmann et al. included 813 patients with type 2 diabetes who were randomized and assigned to semaglutide 1.0 mg weekly versus exenatide ER 2.0 mg weekly [7]. They found greater glycated hemoglobin (HbA1c) reduction (1.5% versus 0.9%) and greater weight loss (5.6 kg versus 1.9 kg). Some gastrointestinal (GI) adverse events were common with semaglutide, including nausea in 22%, vomiting in 7%, and diarrhea in 11%. The main relevance of the trial is the high efficacy of the GLP-1 RA but with a frequent GI intolerance overlapping with perioperative risk [7].

The phase II nonalcoholic steatohepatitis (NASH) trial by Loomba et al. had 108 patients randomized to semaglutide alone or in combination with cilofexor/firsocostat [9]. They reported weight loss of 7%-9.6% and improved hepatic fat and enzymes in combination arms. The GI adverse events were reported in 73%-90% of patients (nausea, 43%; vomiting, 29%). The relevance of the trial confirms that GI intolerance is intrinsic to GLP-1 RA therapy [9].

There is prospective evidence in GLP-1 RA users in which a preoperative gastric ultrasound was performed. It was found that GLP-1 RA users have a significantly higher prevalence of residual gastric contents than controls even after recommended fasting, with the highest risk during dose escalation or when gastrointestinal symptoms were present [10,11]. This supports aspiration risk in plastic surgery.

Some case reports documented perioperative aspiration risk in semaglutide users despite guideline-concordant fasting, underscoring the need for airway protection, selective gastric ultrasound, and individualized perioperative planning [12,13,15-20].

In their meta-analysis, Alves et al. revealed 25 studies of exenatide and liraglutide without finding an increased risk for pancreatitis (OR: 0.87) [21]. They also did not find any overall cancer association. The relevance of this meta-analysis is that it points out rare but uncertain long-term risks that warrant surveillance [15,16,19,21].

In Table 2, we show the comparative evidence of each study.

**Table 2:** Comparative evidence relevant to the perioperative management of GLP-1 RA SUSTAIN-3, Efficacy and Safety of Once-Weekly Semaglutide Versus Exenatide ER in Subjects With Type 2 Diabetes; NASH, nonalcoholic steatohepatitis; T2D, type 2 diabetes; RCT, randomized controlled trial; ER, extended release; HbA1c, glycated hemoglobin; GI AEs, gastrointestinal adverse effects; GLP-1 RA, glucagon-like peptide-1 receptor agonist

Study	Population and design	Intervention	Key efficacy	GI adverse events	Perioperative relevance
Yang and Yang (2024) [1]	T2D; RCT; n=813	Semaglutide versus Exenatide ER	HbA1c reduction, 1.5% versus 0.9%; weight reduction, 5.6 versus 1.9 kg	Nausea, 22%; vomiting, 7%	High efficacy; weekly suspension >1 week
Kommu and Whitfield (2024) [3]	NASH; RCT; n=108	Semaglutide±cilofexor/firsocostat	Weight loss: 7%-9.6%	Nausea, 43%; vomiting, 29%	GI AEs intrinsic to GLP-1; aspiration risk persists
Alves et al. (2012) [21]	25 studies	Liraglutide/exenatide	-	-	No pancreatitis risk; rare cancer signal with liraglutide

Plastic Surgery-Focused Safety Evidence

The multi-society guidance [17] remarks that high-risk patients in elective aesthetic surgery cases should use a 24-hour clear-liquid diet, perform symptom screening, consider selective gastric point-of-care ultrasound (POCUS), and hold weekly GLP-1 RA agents when indicated [16-18].

Sen et al. [10] and Nersessian et al. [11], in their observational evidence results, showed that GLP-1 RA use, especially semaglutide, is associated with increased residual gastric contents, which may pose a risk for aspiration.

In Table 3, we showcase recent studies and guidelines that refine risk stratification for plastic surgery patients using GLP-1 RA.

**Table 3:** Guidance and evidence relevant to plastic surgery safety (2023-2025) RSI, rapid sequence induction; JAMA Surgery, Journal of the American Medical Association of Surgery; APSF, Anesthesia Patient Safety Foundation

Source	Key point	Plastic surgery relevance
Multi-society guidance (2025) [17]	Most can continue with mitigations	Supports individualized management
JAMA Surgery (2024) and Anaesthesia (2024) [10,11]	Increased odds of retained gastric contents	Reinforces aspiration precautions
APSF report and case reports (2023–2024) [12,13,20]	Unexpected solids despite fasting	Necessitates RSI/airway protection

Updated Risk-Stratified Protocol

Different authors suggest that there is a need for a risk-stratified protocol that should consider the screening of the drug, as well as the dose schedule, if the patient is in an escalation phase versus maintenance phase, the presence of GI symptoms suggestive of delayed gastric emptying or even gastroparesis [17,19,20].

For elective cases, if the patient is asymptomatic or in a maintenance dose, the suggestion is to continue with a 24-hour clear-liquid diet and take precautions to avoid aspiration [17].

If the patient undergoing surgery is symptomatic or in a dose escalation regimen, the protocol indicates to hold the administration of the medication, considering whether it is oral dosing or weekly dosing. With daily oral intake, 24-48 hours of suspension is enough; with weekly injections, administration should be stopped more than seven days prior to surgery, and clear liquids and a gastric ultrasound should be added if available [16,17,19].

For the less urgent cases (considering most cases in plastic surgery are elective) or even in an unknown status of GLP-1 RA use, the recommendation is to manage the patient as if they have a full stomach with a rapid sequence intubation and airway protection maneuvers [18,20].

International Guideline Comparison

The 2023 American Society of Anesthesiologists (ASA) guidance was issued in response to early reports of delayed gastric emptying and isolated aspiration events [16]. Based on limited observational evidence and case reports, it recommended a conservative approach, dividing it into days prior to surgery and the day of the surgery management [15,16]. However, it concludes that there is no evidence to suggest the optimal duration of fasting for patients on GLP-1 RA and following the current ASA fasting guidelines [16].

A muti-society guidance statement involving the ASA, American Gastroenterological Association (AGA), American Society for Metabolic and Bariatric Surgery (ASMBS), International Society for the Perioperative Care of Patients with Obesity, and Society of American Gastrointestinal and Endoscopic Surgeons [17] reevaluated the risk-benefit balance and concluded that most patients could continue GLP-1 RA prior to elective surgery, emphasizing the need to apply mitigation strategies and to make an individual risk stratification [17-19]. This information is displayed in Table 4.

**Table 4:** International guideline comparison: ASA (2023) versus multi-society (2025) ASA, American Society of Anesthesiologists; POCUS, point-of-care (gastric) ultrasound

Dimension	ASA (2023) [16]	Multi-society (2025) [17]
Approach	Conservative, universal suspension	Individualized mitigation strategies
Default action	Hold daily agents on the day of surgery; weekly >7 days	Continue in low-risk patients with a 24-hour clear-liquid diet
High risk (symptoms/titration)	Defer surgery; manage as full stomach; consider POCUS	Hold/defer; apply POCUS; 24-hour clear-liquid diet
Proposed tools	Standard fasting; full-stomach management	Symptom screening; selective POCUS; anesthetic adjustments
Plastic surgery relevance	Useful when suspension is feasible without metabolic compromise	Well-suited for an elective context with triage and mitigations

Discussion

Glucose-like peptide-1 receptor agonists (GLP-1 RAs) are transforming the metabolic profile of patients who are candidates for surgery, but their widespread adoption, in some cases without medical indication, has introduced perioperative challenges that are particularly relevant to plastic surgery. Signs of gastrointestinal intolerance, including nausea, vomiting, bloating, and delayed gastric emptying, can occur in up to 40% of patients and represent the principal mechanism related to aspiration risk under general anesthesia [1-3,6,14]. This is particularly significant because in plastic surgery, the procedures involve changing the patient’s position multiple times; i.e., in body contour surgeries, it is not rare to start the surgery in a prone position and switch to a lateral or supine position.

Formulation-specific differences are clinically significant. Daily agents such as liraglutide or oral semaglutide have a shorter half-life and lower systemic persistence. Their discontinuation 24-48 hours before surgery is usually enough in asymptomatic patients. Weekly formulations such as injectable semaglutide, dulaglutide, or tirzepatide can maintain pharmacological activity for several days, needing discontinuation for more than seven days in elective procedures. Most documented cases of retained gastric contents and aspiration involve these long-acting injectables, especially during dose escalation or in patients with gastrointestinal intolerance [10-13,17,19].

Guideline evolution reflects the balance between safety and convenience. ASA 2023 guidelines are based largely on case reports and physiologic sense. They recommend a conservative approach: universal suspension (day of surgery for daily formulations; >7 days for weekly formulations). However, to increase safety and lower the risk of perioperative aspiration, we agree with some authors that even daily oral or subcutaneous (SC) injections should be suspended at least seven days before surgery. This approach maximizes safety but increases the risk of metabolic decompensation and logistic challenges, and therefore, a consultation with the endocrinologist can be made.

In contrast, the October 2024 multi-society guidance (ASA, AGA, ASMBS, and others) endorsed a risk-stratified approach with continuation of the drug in most patients, with some mitigations such as a 24-hour clear-liquid diet, symptom screening, selective gastric ultrasound if available, and suspension or deferral only in high-risk scenarios. In plastic surgery, where most cases are elective, this risk-based framework is especially convenient, as it allows to individualize by drug type, dosing status, and patient characteristics [16-19].

Prospective evidence has shifted the debate from theoretical to clinical demonstration. In a prospective study using gastric ultrasound, GLP-1 RA users had a higher prevalence of residual gastric contents (56% versus 19%); a second prospective observational study reported 40% versus 3% with recent semaglutide exposure. Additional case reports describe aspiration despite guideline-concordant fasting. Together, these findings support structured checklists, selective gastric POCUS, and standardized algorithms to improve safety [10-13,20].

Implications for plastic surgery are not distant. Aesthetic and reconstructive procedures often involve prolonged general anesthesia, and patients with obesity are more susceptible to aspiration risk or difficult airway management. This is especially true in body contouring surgery, where not only is the patient in a supine position but also multiple intraoperative position changes (supine, prone, lateral, and jackknife) may occur within the same procedure, further complicating airway protection and anesthesia management. By contrast, facial and breast procedures are shorter and generally pose a lower risk, although rigorous preoperative screening remains essential [17-20].

We believe that plastic surgeons should incorporate the evaluation of drug, formulation, dosing schedule, escalation status, GI symptoms, and psychiatric history, including screening for suicidal ideation, as recently reported with some GLP-1 RA, into the standard preoperative workflow.

In the context of elective and emergency plastic surgery, the emerging reports linking glucagon-like peptide-1 receptor agonists (GLP-1 RAs) with mood alterations and, in rare cases, suicidal ideation warrant careful consideration. Because these agents modulate central neuroendocrine pathways involved in appetite regulation, reward processing, and stress responses, their potential neuropsychiatric effects may influence perioperative safety and patient outcomes. For elective procedures, the early identification of mood disturbances allows for the postponement of surgery until psychological stability is ensured, thereby improving adherence to perioperative instructions and reducing the likelihood of postoperative complications. In urgent reconstructive scenarios, continued vigilance remains essential: the prompt recognition of suicidal thoughts facilitates timely psychiatric intervention and supports informed, ethically sound decision-making. Incorporating routine mental health screening into the preoperative assessment of patients using GLP-1 RAs reinforces a multidisciplinary framework that prioritizes patient safety and aligns with current standards of comprehensive surgical care.

High-risk patients, such as those in weekly formulations, undergoing dose escalation, with GI symptoms or gastroparesis evidence, and even those with psychiatric red flags, should undergo discontinuation protocols. The suggestion is >7 days for weekly formulations and 24-48 hours for daily formulations, and anesthesiologists should manage these patients as if they had a full stomach. In low-risk patients such as those taking daily agents, undergoing a stable or maintenance dose, and GI asymptomatic, we can safely proceed with shorter holds or even the continuation of drugs if mitigation strategies are applied [14,15,17,19].

There was some concern about the use of GLP-1 RA and pancreatitis. However, there are meta-analyses that have not demonstrated increased pancreatitis risk but raise possible cancer signals with liraglutide that will require vigilance. Beyond anesthesia and nutritional and psychiatric safety, some domains are emerging micronutrient deficiencies, and more reports of suicidal ideation underscore the need for comprehensive perioperative evaluation. While causality remains uncertain, this highlights the need for a comprehensive preoperatory evaluation that includes both physical and psychological risk factors.

Moreover, most available data come from endocrinology, hepatology, and anesthesiology, with limited plastic surgery-specific outcomes. There is a pressing need for prospective, specially driven studies to quantify aspiration, nausea, vomiting, wound healing, and metabolic outcomes after temporary drug suspension [14,15,17,19,21].

It is a reality that GLP-1 RAs are here to stay in everyday surgical practice. Plastic surgeons must take a leadership role in designing tailored risk-stratified perioperative protocols that integrate pharmacology, anesthesiology, endocrinology, nutrition, and psychiatry [7,9,16,17]. The convergence of randomized trials, prospective studies, case reports, and evolving guidelines supports a hybrid model: conservative for high-risk patients, permissive with mitigations for low-risk patients, and universally grounded in multidisciplinary coordination [4-7,9,16,19-21]. Structured checklists, perioperative algorithms, and prospective registries will be key to ensuring safety while maintaining the metabolic and aesthetic benefits of these therapies [1,2,10-13,16,17,19,21].

**Figure 1:**
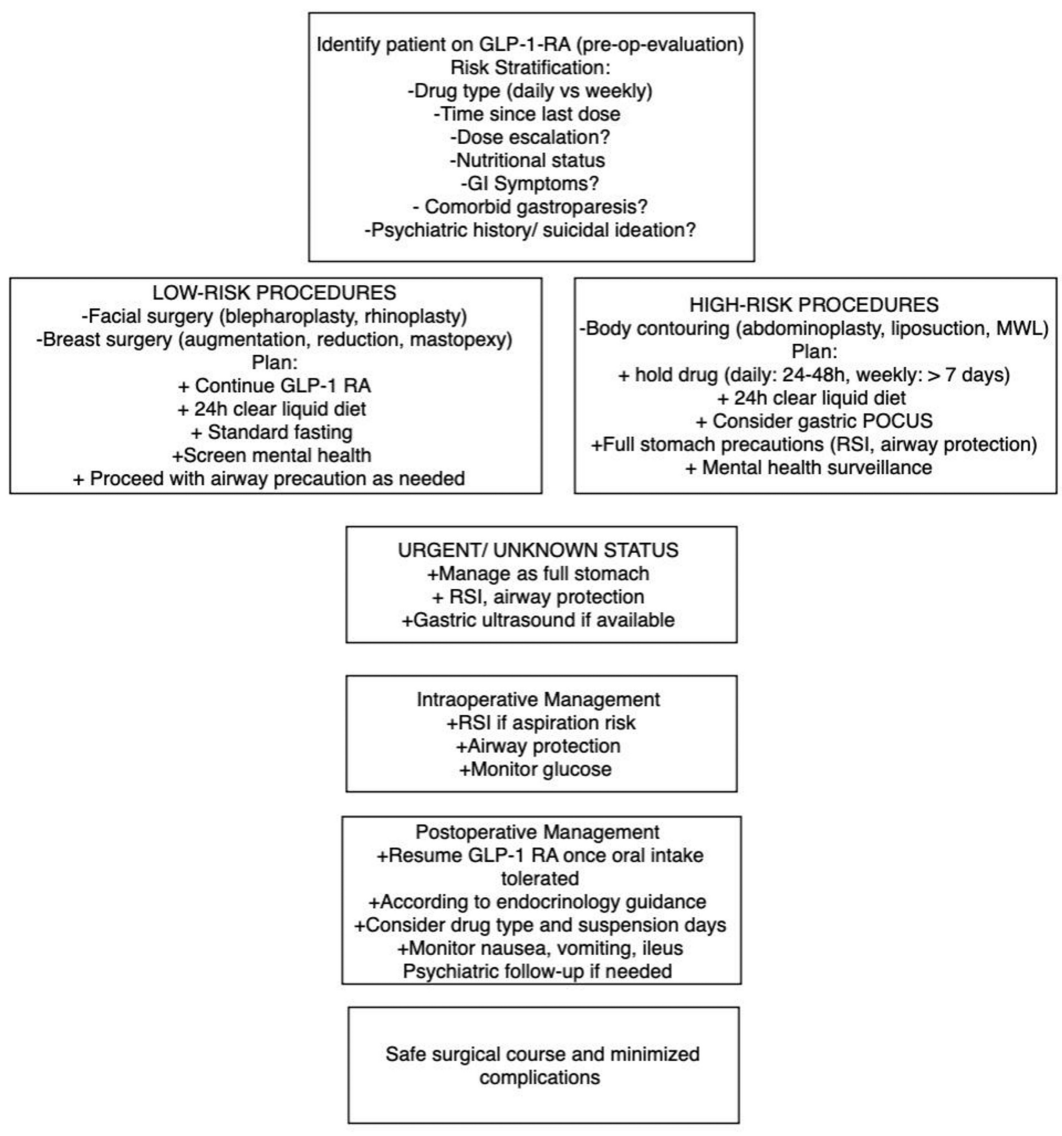
Perioperative checklist for GLP-1 RA therapy GLP-1 RA, glucose-like peptide-1 receptor agonist; GI, gastrointestinal; MWL, massive weight loss; POCUS, point-of-care ultrasound; RSI, rapid sequence intubation

Taking into consideration all the evidence, we propose a preoperative, intraoperative, and postoperative checklist, as seen in Figure 1, to safely manage patients taking GLP-1 RA and undergoing elective or even urgent plastic surgery [10,11,16-20].

## Conclusions

Glucagon-like peptide-1 receptor agonists (GLP-1 RAs) are commonly used among plastic surgery patients, and their use may grow exponentially over the next few years. There is a concern in some of their pharmacological effects, particularly delayed gastric emptying and gastrointestinal intolerance, that pose a unique perioperative, transoperative, and postoperative challenge that demands proactive management from the plastic surgery team. We are aware that we conducted a narrative review rather than a systematic review under Preferred Reporting Items for Systematic Reviews and Meta-Analyses (PRISMA) guidelines, and this can be a limitation. We will consider this for future projects.

GLP-1 RA represents both a therapeutic opportunity and a surgical challenge: they improve metabolic health yet impose novel anesthetic and psychiatric risks. Plastic surgeons must lead the development of tailored, evidence-based, risk-stratified protocols that safeguard patients while preserving the benefits of these therapies. We are confident that future plastic surgery-specific prospective studies and registries are urgently needed to validate these strategies, quantify complication rates, and refine best practices in the era of metabolic pharmacology.
